# Deep-learning-based survival prediction of patients with lower limb melanoma

**DOI:** 10.1007/s12672-023-00823-y

**Published:** 2023-11-30

**Authors:** Jinrong Zhang, Hai Yu, Xinkai Zheng, Wai-kit Ming, Yau Sun Lak, Kong Ching Tom, Alice Lee, Hui Huang, Wenhui Chen, Jun Lyu, Liehua Deng

**Affiliations:** 1 Department of Dermatology, The First Affiliated Hospital of Jinan University and Jinan University Institute of Dermatology, Guangzhou, 510630 China; 2 Department of Infectious Diseases and Public Health, Jockey Club College of Veterinary Medicine and Life Sciences, City University of Hong Kong, Hong Kong, China; 3 Centro de Hospitalar Conde de Januario, Macau, China; 4 Primax Biotech Company, Hong Kong, China; 5 Hong Kong Medical and Education, Hong Kong, China; 6 Shanghai Aige Medical Beauty Clinic Co., Ltd. (Agge), Shanghai, China; 7 Department of Clinical Research, The First Affiliated Hospital of Jinan University, Guangzhou, China; 8 Guangdong Provincial Key Laboratory of Traditional Chinese Medicine Informatization, Guangzhou, China; 9 Department of Dermatology, The Fifth Affiliated Hospital of Jinan University, Heyuan, China

**Keywords:** DeepSurv, Lower limb melanoma, Neural network, Survival prediction, SEER

## Abstract

**Background:**

For the purpose to examine lower limb melanoma (LLM) and its long-term survival rate, we used data from the Surveillance, Epidemiology and End Results (SEER) database. To estimate the prognosis of LLM patients and assess its efficacy, we used a powerful deep learning and neural network approach called DeepSurv.

**Methods:**

We gathered data on those who had an LLM diagnosis between 2000 and 2019 from the SEER database. We divided the people into training and testing cohorts at a 7:3 ratio using a random selection technique. To assess the likelihood that LLM patients would survive, we compared the results of the DeepSurv model with those of the Cox proportional-hazards (CoxPH) model. Calibration curves, the time-dependent area under the receiver operating characteristic curve (AUC), and the concordance index (C-index) were all used to assess how accurate the predictions were.

**Results:**

In this study, a total of 26,243 LLM patients were enrolled, with 7873 serving as the testing cohort and 18,370 as the training cohort. Significant correlations with age, gender, AJCC stage, chemotherapy status, surgery status, regional lymph node removal and the survival outcomes of LLM patients were found by the CoxPH model. The CoxPH model’s C-index was 0.766, which signifies a good degree of predicted accuracy. Additionally, we created the DeepSurv model using the training cohort data, which had a higher C-index of 0.852. In addition to calculating the 3-, 5-, and 8-year AUC values, the predictive performance of both models was evaluated. The equivalent AUC values for the CoxPH model were 0.795, 0.767, and 0.847, respectively. The DeepSurv model, in comparison, had better AUC values of 0.872, 0.858, and 0.847. In comparison to the CoxPH model, the DeepSurv model demonstrated greater prediction performance for LLM patients, as shown by the AUC values and the calibration curve.

**Conclusion:**

We created the DeepSurv model using LLM patient data from the SEER database, which performed better than the CoxPH model in predicting the survival time of LLM patients.

**Supplementary Information:**

The online version contains supplementary material available at 10.1007/s12672-023-00823-y.

## Introduction

Lower limb melanoma is a challenging and potentially life-threatening condition, and accurate survival prediction is crucial for guiding treatment decisions and improving patient outcomes [1]. The lower limbs encompass the entire region from the hips down to the feet, making them the largest area of skin surface on the human body. While this increased surface area offers more opportunities for melanoma to develop [2, 3].

DeepSurv, a powerful deep learning algorithm designed for time-to-event analysis, holds immense potential in revolutionizing the prediction of survival rates for individuals diagnosed with lower limb melanoma [4].

Melanoma, arising from the uncontrolled growth of melanocytes, can significantly impact a patient’s life, making early and accurate survival predictions paramount in tailoring personalized treatment plans [5, 6]. Conventional survival analysis methods often rely on statistical models that assume linear relationships between covariates and survival times, which might not capture the complex, non-linear patterns present in melanoma progression [7]. DeepSurv, on the other hand, overcomes these limitations by leveraging deep neural networks to effectively capture intricate relationships between various factors and time-to-event outcomes [4].

The lower limbs represent a challenging area for melanoma prognosis, as they can exhibit diverse clinical presentations and complex biological behaviors [8]. DeepSurv can analyze extensive patient data, encompassing demographic information, medical history, and genetic profiles, to provide a comprehensive and accurate assessment of a patient’s prognosis [9]. By learning from large datasets of lower limb melanoma cases, DeepSurv can identify subtle patterns and risk factors that may influence the survival outcome, thus improving the accuracy of predictions [4].

Moreover, DeepSurv is well-suited to continuously adapt and refine its predictions as new data becomes available [10]. As medical research advances and more lower limb melanoma cases are recorded, the algorithm can seamlessly incorporate this information into its learning process, ensuring that predictions remain up-to-date and reflective of the latest medical knowledge [11].

By employing DeepSurv for lower limb melanoma survival analysis, clinicians can make more informed decisions when determining the most appropriate treatment strategies for individual patients [12]. This tailored approach may lead to improved patient outcomes, reduced treatment-related adverse effects, and a higher overall quality of life for those affected by this aggressive form of skin cancer [13].

## Materials and methods

### Data filtering criteria

**Fig. 1:**
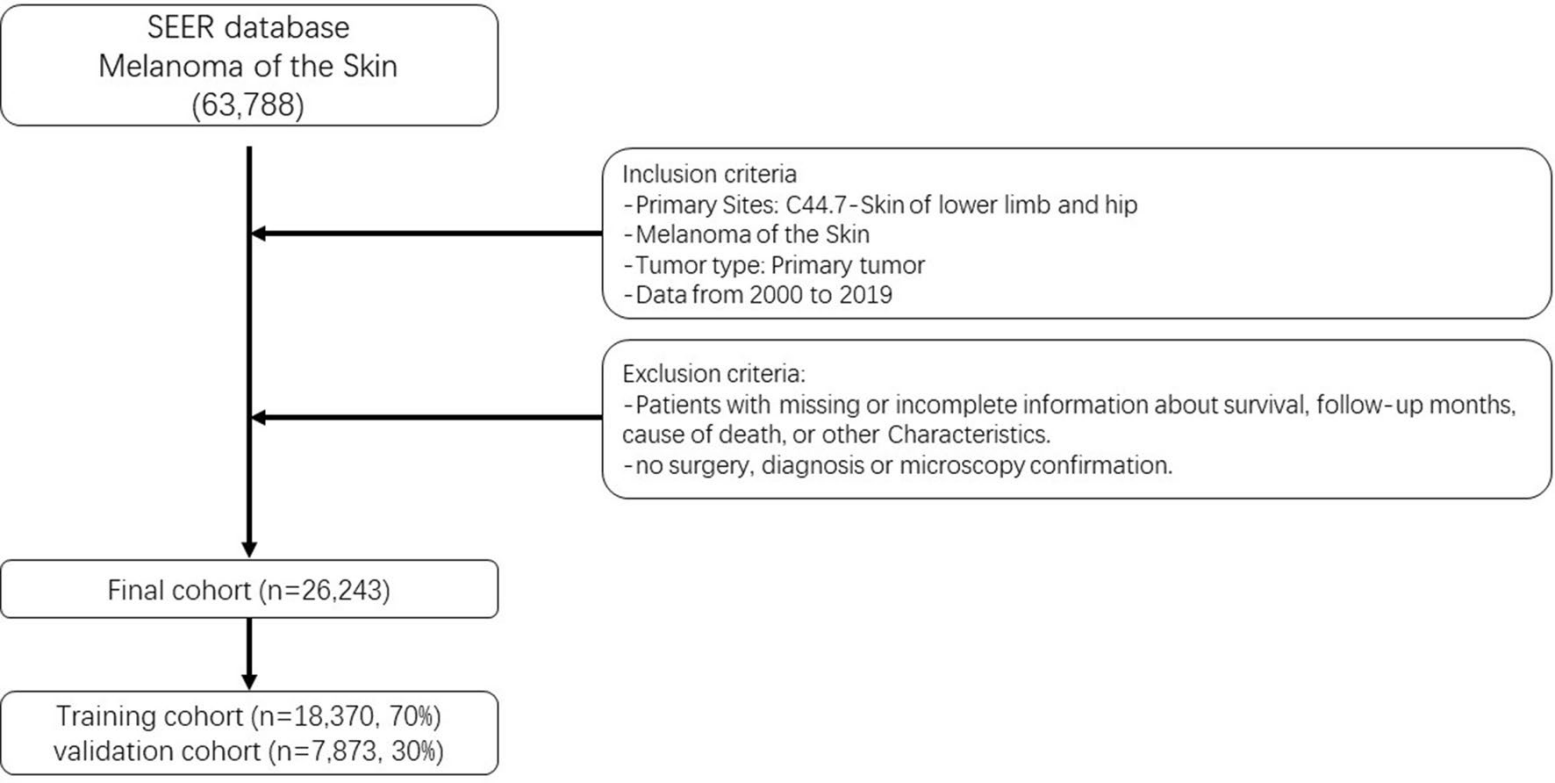
Flow chart of patient selection

The SEER*Stat software, version 8.4.1, was employed to meticulously examine patients afflicted with Lower Limb Melanoma (LLM) [14, 15]. Incidences of melanoma in this category were curated by applying the histology/behavior codes as outlined in the third revision of the International Classification of Diseases for Oncology (ICD-O-3), specifically under the classification “Melanoma of the Skin.” Moreover, cases pertaining to the region designated as “C44.7-Skin of lower limb and hip” were judiciously selected for the purpose of analysis. Patients exhibiting nonprimary tumors and those with insufficient foundational data were systematically excluded from the ensuing analysis. Ultimately, a comprehensive cohort of 26,243 LLM patients was encompassed within this meticulous investigation, spanning the period from 2000 to 2019. It is important to underscore that neither ethical committee endorsement nor formal written consents were deemed requisite, as the entirety of the data employed from the SEER database, barring patient identification particulars, remains fully accessible to the general populace. Figure 1 artfully portrays the schematic representation of the patient selection process.

### Patient information classification criteria

The variables subjected to meticulous examination encompassed a myriad of factors, spanning age, gender, ethnicity, marital status, tumor dimensions, tumor dissemination, TNM stage, summary stage, surgical intervention, administration of radiotherapy, chemotherapy treatment, lymph node dissection subsequent to surgery (Reg LN Sur), primary site surgical intervention, sequence of radiotherapy (Rad Seq), and income. Notably, the median duration of follow-up for this investigation extended to an impressive 92 months, ranging from 1 to 191 months. Regarding racial demographics, participants were stratified into three distinct categories: White, Black, and Other, while marital status entailed categorizations of Married, Single, and Other. Reg LN Sur was thoughtfully compartmentalized into three distinct classifications: lymph node removal, absence of lymph node inspection, and postoperative lymph node dissection. The various categories for Rad Seq encompassed No radiation, radiotherapy before surgery, radiotherapy during surgery, radiotherapy after surgery, and radiotherapy both before and after surgery.

### DeepSurv model design

**Fig. 2:**
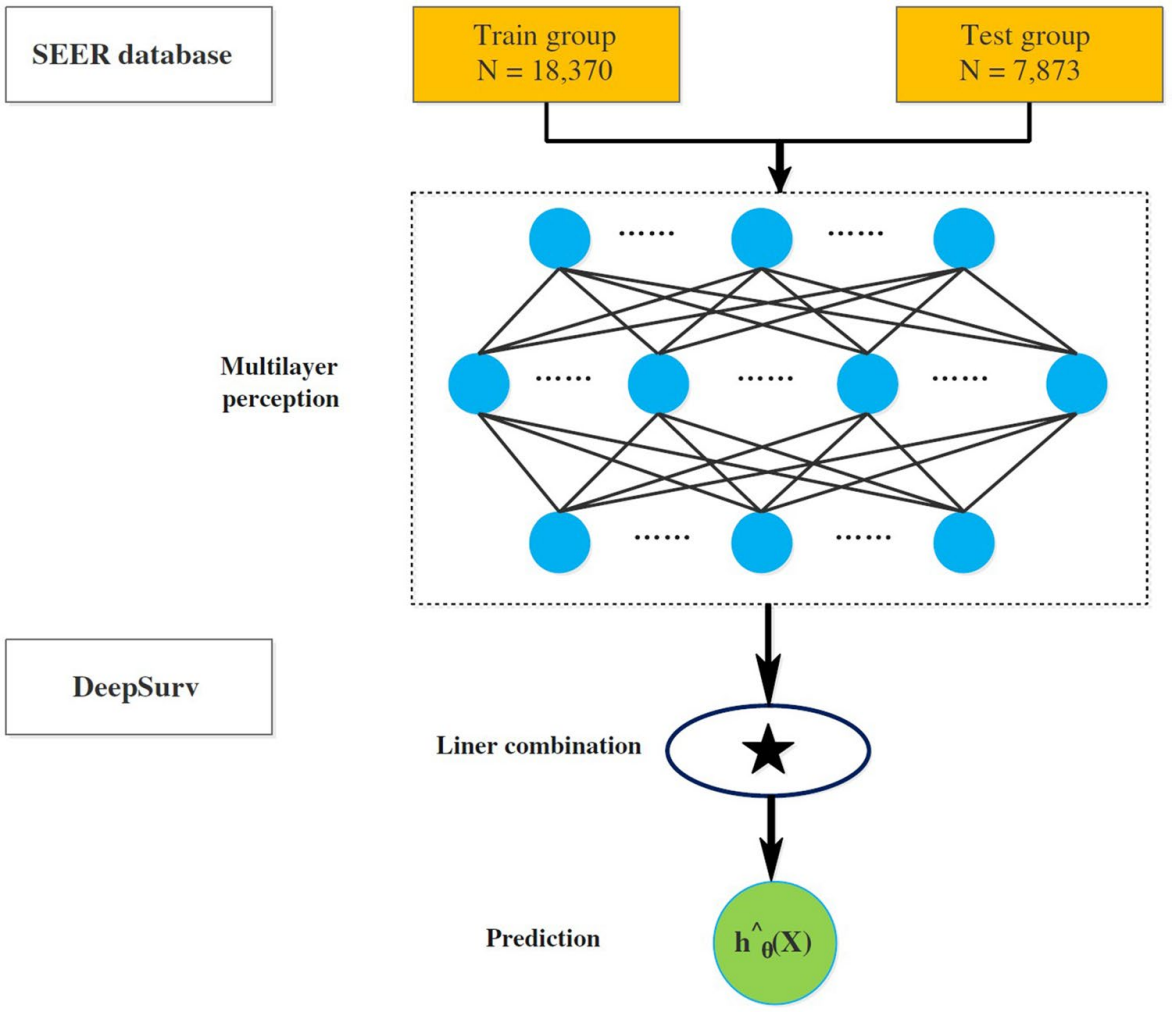
Diagram of the deep learning procedure

The DeepSurv model, an intricately designed feedforward neural network, is composed of three layers: the input layer, the hidden layer, and the output layer. This sophisticated model harnesses a multitude of simulated neurons to intricately process the data at hand. The input layer of the DeepSurv model primarily comprises the foundational patient data (x). Moving forward, the hidden layer incorporates a fully connected nonlinear activation function, dropout regularization, and an additional array of hidden units. Finally, the output layer, denoted as h^θ(x) [4] (as depicted in Fig. 2), yields the estimated risk value. Our implementation of the model, meticulously crafted using the PyTorch deep learning framework, heavily relies on pycox for seamless execution of neural network computations. By leveraging pertinent clinical characteristics, our model adeptly predicts the impact on patient survival and generates a corresponding risk value.

To thoroughly assess the model’s performance, we bifurcated the LLM patients into distinct training and testing groups. Employing the training cohort data, we diligently constructed an initial DeepSurv model, carefully architecting it with a neural network comprising seven meticulously crafted layers. Subsequently, this well-constructed model was deployed to conduct comprehensive survival analysis on the LLM patients within the designated testing cohort. In order to evaluate the model’s discrimination, calibration, and overall efficacy, an array of evaluation metrics was employed, including the concordance index (C-index), calibration curve, and receiver operating characteristic (ROC) curve [16]. These meticulously chosen metrics effectively served as benchmarks for comparative analysis, providing invaluable insights into the performance of the DeepSurv models.

### Statistical analysis

Categorical variables were eloquently presented as proportions, while continuous variables were succinctly summarized using the median and interquartile range. The CoxPH model was meticulously crafted utilizing R (version 4.2.0), showcasing its statistical prowess. On the other hand, the DeepSurv model was artfully developed employing Python (version 3.8.0), taking advantage of the dynamic capabilities of this programming language. The Python ecosystem, bolstered by the versatile pandas, visually appealing matplotlib.pyplot, and efficient NumPy modules, adeptly facilitated data calculations, model training, and various other operations. For the construction of the deep learning neural network within the DeepSurv model, the esteemed PyTorch framework and the pycox module were thoughtfully employed, attesting to the attention to detail in the model implementation [17]. Furthermore, statistical significance was judiciously defined as p < 0.05, aligning with the established conventions of rigorous scientific inquiry.

## Results

### Basic information of patients

The study encompassed an extensive cohort of 26,243 patients diagnosed with Lower Limb Melanoma (LLM), meticulously segregated into two distinct cohorts: a training cohort comprising 18,370 patients (70%), and a testing cohort comprising 7873 patients (30%). Among the patient population, 8370 individuals (31.92%) were male, while 17,865 (68.08%) were female, highlighting a notable gender distribution. The average age of the patients was reported as 57.61 years, indicating a mature and diverse population, with a noteworthy majority of 25,370 individuals (96.67%) identifying as of white ethnicity, signifying the predominant racial composition.

Regarding disease staging, a significant proportion of patients were classified as T1 (15,998, 60.96%), N0 (22,763, 86.74%), and M0 (25,677, 97.84%), underlining the early stage and localized nature of the majority of cases. The median duration of follow-up encompassed 92 months, with a range spanning from 1 to 191 months, showcasing a substantial observation period capturing diverse patient trajectories. The cumulative number of deaths attributed to LLM amounted to 6367 cases (24.26%), shedding light on the gravity of this condition.

**Fig. 3:**
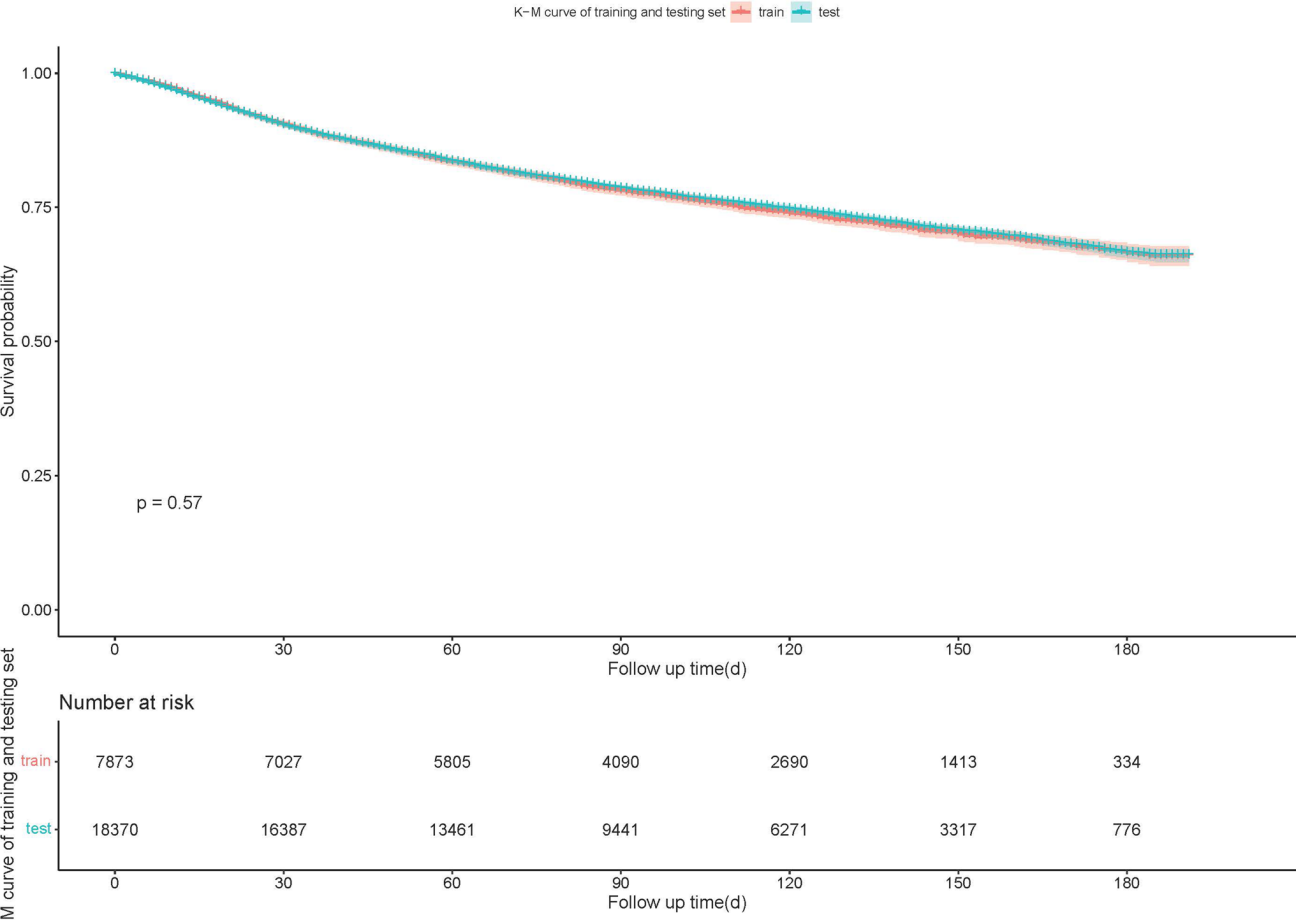
Kaplan–Meier curve of training and testing cohort. There was no statistically significant difference between the survival of the training and testing cohort in the log-rank test (*p* = 0.57)

Notably, the survival curves and essential clinical data demonstrated no notable disparities between the two cohorts, affirming the appropriate division and balance of the patient groups. For a comprehensive overview of the fundamental characteristics exhibited by the patient groups, please refer to Table 1, while Fig. 3 elegantly portrays the Kaplan–Meier analysis curve, providing a visual representation of survival trends in this LLM cohort.

**Table 1:** Baseline Characteristics

Variable	Total N (%)	Train cohort N (%)	Test cohort N (%)
Total	26,243	18,370	7873
Age			
Mean ± SD	57.61 ± 16.74	57.62 ± 16.75	57.6 ± 16.71
CS_tumor_size^a^			
Mean ± SD	158.1 ± 485.12	578.9 ± 485.47	586.3 ± 484.29
CS_extension^b^			
Mean ± SD	253 ± 138.98	253.5 ± 138.44	251.9 ± 140.23
Surg_Prim_Site^c^			
Mean ± SD	36.62 ± 9.92	36.66 ± 9.92	36.52 ± 9.92
Sex			
Male	8378 (31.92%)	5860 (31.9%)	2518 (31.98%)
Female	17,865 (68.08%)	12,510 (68.1%)	5355 (68.02%)
Race			
White	25,370 (96.67%)	17,782 (96.8%)	7588 (96.38%)
Black	369 (1.41%)	244 (1.33%)	125 (1.59%)
Other	504 (1.92%)	344 (1.87%)	160 (2.03%)
Marital			
Single	4382 (16.7%)	3005 (16.36%)	1377 (17.49%)
Married	17,263 (65.78%)	12,125 (66%)	5138 (65.26%)
DSW	4598 (17.52%)	3240 (17.64%)	1358 (17.25%)
AJCC			
I	18,859 (71.86%)	13,246 (72.11%)	5613 (71.29%)
II	3739 (14.25%)	2527 (13.76%)	1212 (15.39%)
III	3079 (11.73%)	2197 (11.96%)	882 (11.2%)
IV	566 (2.16%)	400 (2.18%)	166 (2.11%)
T			
T0	34 (0.13%)	26 (0.14%)	8 (0.1%)
T1	15,998 (60.96%)	11,214 (61.05%)	4784 (60.76%)
T2	4783 (18.23%)	3377 (18.38%)	1406 (17.86%)
T3	2967 (11.31%)	2055 (11.19%)	912 (11.58%)
T4	2044 (7.79%)	1412 (7.69%)	632 (8.03%)
TX	417 (1.59%)	286 (1.56%)	131 (1.66%)
N			
N0	22,763 (86.74%)	15,895 (86.53%)	6868 (87.23%)
N1	1829 (6.97%)	1284 (6.99%)	545 (6.92%)
N2	978 (3.73%)	717 (3.9%)	261 (3.32%)
N3	598 (2.28%)	420 (2.29%)	178 (2.26%)
NX	75 (0.29%)	54 (0.29%)	21 (0.27%)
M			
M0	25,677 (97.84%)	17,970 (97.82%)	7707 (97.89%)
M1	566 (2.16%)	400 (2.18%)	166 (2.11%)
Summary_Stage			
Localized	21,981 (83.76%)	15,355 (83.59%)	6626 (84.16%)
Regional	3544 (13.5%)	2510 (13.66%)	1034 (13.13%)
Distant	718 (2.74%)	505 (2.75%)	213 (2.71%)
Surgery			
Yes	25,699 (97.93%)	17,997 (97.97%)	7702 (97.83%)
No	544 (2.07%)	373 (2.03%)	171 (2.17%)
Radiation			
Yes	384 (1.46%)	265 (1.44%)	119 (1.51%)
No	25,859 (98.54%)	18,105 (98.56%)	7754 (98.49%)
Chemotherapy			
Yes	541 (2.06%)	391 (2.13%)	150 (1.91%)
No	25,702 (97.94%)	17,979 (97.87%)	7723 (98.09%)
Reg_LN_Sur^d^			
Yes	12,615 (48.07%)	8850 (48.18%)	3765 (47.82%)
No	13,628 (51.93%)	9520 (51.82%)	4108 (52.18%)
Rad_Seq^e^			
Before	5 (0.02%)	4 (0.02%)	1 (0.01%)
After	275 (1.05%)	206 (1.12%)	69 (0.88%)
Intraoperative	1 (0%)	0 (0%)	1 (0.01%)
Both	3 (0.01%)	2 (0.01%)	1 (0.01%)
No	25,959 (98.92%)	18,158 (98.85%)	7801 (99.09%)
Income			
Low	4383 (16.7%)	3095 (16.85%)	1288 (16.36%)
Mediate	12,432 (47.37%)	8670 (47.2%)	3762 (47.78%)
High	9428 (35.93%)	6605 (35.96%)	2823 (35.86%)

^a^CS tumor size: Information on tumor size. Available for after 2004 year. Earlier cases may be converted and new codes added which weren’t available for use prior to the current version of CS

^b^CS extension: Information on extension of the tumor. Available for after 2004 year. Earlier cases may be converted and new codes added which weren’t available for use prior to the current version of CS

^c^Surg_Prim_Site: Surgery of Primary Site describes a surgical procedure that removes and/or destroys tissue of the primary site performed as part of the initial work-up or first course of therapy

^d^Reg_LN_Sur: Scope of Regional Lymph Node Surgery describes the procedure of removal,biopsy, or aspiration of regional lymph nodes performed during the initial work-up or first course of therapy at all facilities

^e^Rad_Seq: This field records the order in which surgery and radiation therapies were administered for those patients who had both surgery and radiation

### Variable screening and DeepSurv model training

Through the meticulous implementation of the CoxPH model on the training cohort, a comprehensive analysis of the multivariate factors was conducted, discerning influential risk factors contributing to patient mortality. These factors encompassed age, sex, AJCC (American Joint Committee on Cancer) stage, surgery status, chemotherapy status, and Reg_LN_Sur (regional node biopsy), as eloquently presented in Table 2. Notably, the CoxPH model exhibited a commendable C-index of 0.766, reflecting its ability to discriminate and predict outcomes with a high level of accuracy.

**Table 2:** Survival predictors in Cox PH model

Variables	β	HR	95%CI	*p*
Age	0.06444	1.067	1.065–1.069	< 0.0001
Sex female	− 0.32679	0.721	0.686–0.758	< 0.0001
AJCC II	1.10078	3.007	2.811–3.215	< 0.0001
AJCC III	1.71754	5.571	5.174–5.998	< 0.0001
AJCC IV	2.47833	11.921	10.681–13.306	< 0.0001
Surgery no	0.65681	1.929	1.699–2.189	< 0.0001
Chemotherapy no	− 0.6691	0.512	0.455–0.576	< 0.0001
Reg_LN_Sur no	0.19747	1.218	1.148–1.293	< 0.0001

**Fig. 4:**
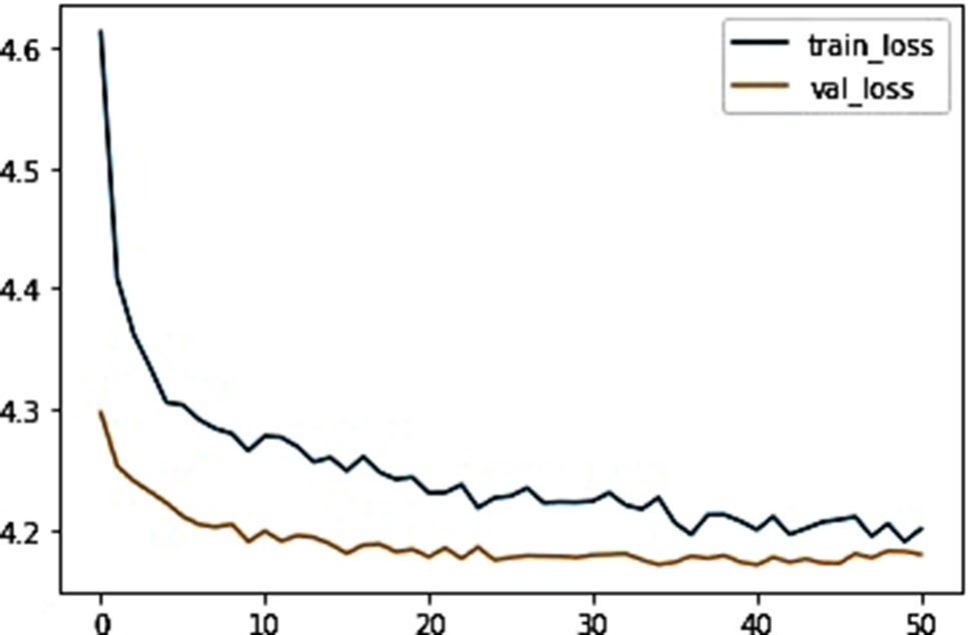
The loss change process diagram of training and validating. train_loss: train loss; Val_loss: validation loss. Train loss is the loss on the training data, which measures the fitting ability of the model on the training set. Val loss is the loss on the validation set, which measures the fitting ability on unseen data

In parallel, the successful construction of the DeepSurv model, utilizing the training cohort, yielded a remarkable C-index of 0.852. The superiority of the DeepSurv model over the CoxPH model in terms of efficacy is readily apparent. This enhanced performance is visually depicted through the training loss-function diagram, artfully presented in Fig. 4. The diagram stands as a testament to the DeepSurv model’s robust performance, showcasing its proficiency in effectively capturing and interpreting complex survival patterns, thus enhancing the overall predictive capacity of the model.

### Comparison of the DeepSurv model and CoxPH model in the testing cohort

**Fig. 5:**
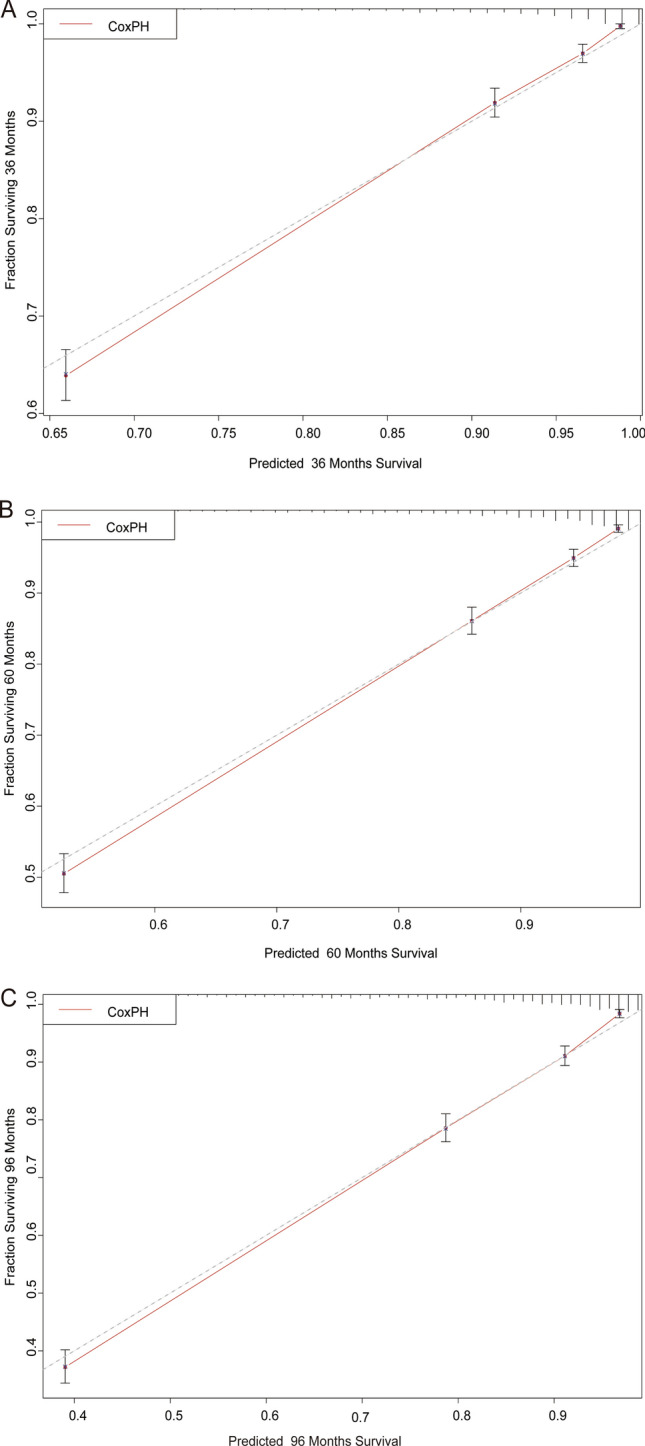
Calibration plots of the survival rate of LLM in the Cox PH model. **A** 3 Years of the survival rate. **B** 5 Years of the survival rate. **C** 8 Years of the survival rate

**Fig. 6:**
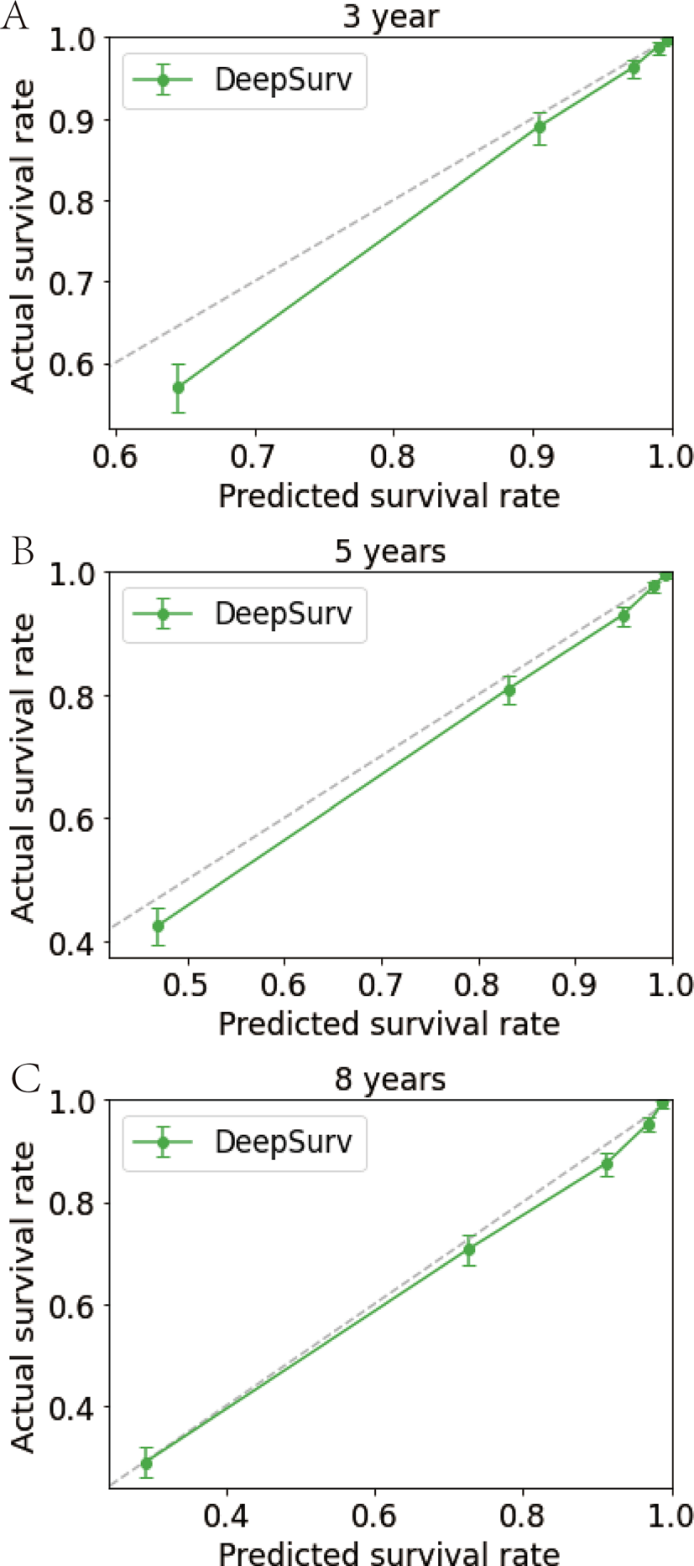
Calibration plots of survival rate of LLM in DeepSurv model. **A** 3 Years of the survival rate. **B** 5 Years of the survival rate. **C** 8 Years of the survival rate

**Fig. 7:**
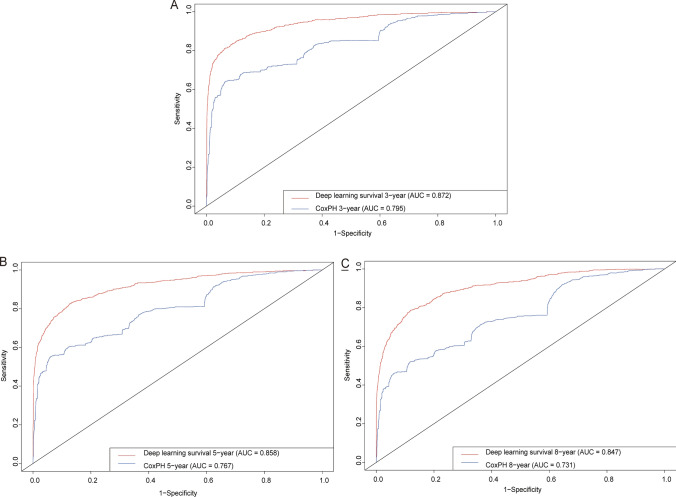
ROC curves. Comparison of ROC between the CoxPH model and the DeepSurv model in 3 year (**A**), 5 year (**B**), and 8 year (**C**)

To rigorously assess the precision and reliability of both the CoxPH model (Fig. 5) and the DeepSurv model (Fig. 6) in estimating survival probability, we proceeded to construct calibration curves for patients diagnosed with LLM at 3, 5, and 8 years. These calibration curves allow for a visual comparison of the predicted and observed survival probabilities, providing insights into the models’ calibration performance. Additionally, the discrimination between the two models can be evaluated by plotting ROC curves for the LLM patients at 3, 5, and 8 years into the future. Notably, the time-dependent area under the ROC curve (AUC) value can be calculated to quantify and compare their discrimination performance (Fig. 7).

The results unequivocally demonstrate that the DeepSurv model exhibits superior performance in comparison to the CoxPH model. This is evidenced by the higher AUCs at 3, 5, and 8 years (0.872, 0.858, and 0.847, respectively) for the DeepSurv model, surpassing the AUCs of the CoxPH model (0.795, 0.767, and 0.847, respectively). These findings conclusively establish the DeepSurv model’s enhanced prognostic accuracy and calibration capabilities in predicting the survival prognosis of patients with LLM.

In summary, the calibration curves and ROC curves provide compelling evidence of the DeepSurv model’s superiority over the CoxPH model, offering a more precise and reliable estimation of survival probability for patients diagnosed with LLM at various time points in the future. This robust performance of the DeepSurv model underscores its potential to serve as a valuable tool for clinical prognostication and patient management in the context of LLM.

## Discussion

Melanoma, the most aggressive form of skin cancer, poses a significant health challenge worldwide [18]. Among the various types of melanoma, lower limb melanoma accounts for a substantial proportion of cases [19]. Accurate prognosis and survival estimation are crucial for guiding treatment decisions and improving patient outcomes [20]. Deep learning techniques, such as DeepSurv, have shown promise in predicting patient survival rates based on clinical and genetic features.

This study explores the potential of DeepSurv in advancing our understanding of lower limb melanoma and its implications for personalized medicine. Deep learning model specifically designed for survival analysis [4]. Unlike traditional statistical methods, which assume cox proportional hazards, DeepSurv is capable of handling complex, high-dimensional data and non-linear relationships [21]. It predicts survival probabilities over time, enabling precise and dynamic risk assessments for patients. It also can leverage diverse data sources, including clinical records, histopathological data, and genetic profiles. Integrating this information can provide a more comprehensive picture of the patient’s condition and potential risk factors [22, 23].

Traditional prognostic models in melanoma often rely on a limited set of variables, leading to generalized estimates [7, 24]. DeepSurv, with its ability to capture complex relationships, may offer more precise and individualized survival predictions for patients, helping oncologists tailor treatments to specific needs.

Within the CoxPH model, a variety of factors such as age, sex, AJCC, surgical interventions, chemotherapy, and Reg_LN_Sur were identified as significant risk factors impacting the domain of LLM. Furthermore, the CoxPH model exhibited good C-index, attesting to its commendable predictive precision.

DeepSurv’s capacity to analyze vast amounts of data may lead to the discovery of novel prognostic markers for lower limb melanoma. These markers could unlock new avenues for targeted therapies and early intervention strategies. And it also accounts for the timing of events, providing time-dependent survival probabilities. This capability is particularly relevant in melanoma, where the disease progression can vary over time.

So, the newly developed DeepSurv model, consisting of an intricate neural network with multiple discerning layers, achieved remarkable performance with a higher C-index, which is 0.852. Notably, there was a noticeable disparity in the calibration curves between the DeepSurv and CoxPH models. The DeepSurv model demonstrated a more evenly distributed profile, aligned harmoniously with the leading-diagonal line, which indicated its superiority. This superiority was further evident in the AUC curve, where the DeepSurv model exhibited exceptional smoothness that surpassed its CoxPH counterpart, reaffirming its prowess in predicting 3-, 5-, and 8-year mortality and survival-time outcomes for patients with LLM [16]. The reason we choose 3-, 5-, and 8-year mortality is due to previous research. Lower limb melanoma survival may have identified these time points as important for assessing long-term outcomes or for making comparisons with other studies [25, 26]. Also, in cancer stat facts of melanoma (https://seer.cancer.gov/statfacts/html/melan.html) the 5-year survival is 93.5%. Therefore, we collected data and made observations both before and after the 5 years survival.

The correlations of our deep learning results with those of other authors in the field provides essential validation and contextualization of our findings [22, 23, 27, 28]. It allows us to gauge the generalizability and clinical utility of our model, identify potential challenges, and highlight its strengths in specific patient populations or cancer types. By embracing collaboration and comparison across studies, we can collectively advance the field of deep learning for cancer and foster its seamless integration into clinical practice for improved patient outcomes.

The DeepSurv model’s predictions have several values in healthcare application. For high-risk patients’ intensive treatments or closer monitoring should be able identified by the doctors which will give to customize treatment plans, while low-risk patients be able to use fewer intensive treatments, for a better cause. Also, in clinical daywork we face limited resources, with DeepSurv model’s we can allocate the appropriate amount resources for whom needs it, this way we can raise the efficiency. Make sure high-risk patients would receive appropriate follow-up and specialized care so clinicians can offer patients with precise information, facilitating the right prognosis and treatment plans. Moreover, for patients with better outcomes, the DeepSurv model’ can provide with more accurate long-term care plans, with monitoring the recurrence, and other life matter concerns. Based on the result of each model, for cancer epidemiology, trends, and survival outcomes we still can use SEER database to give us the insight based on what SEER database has.

This study has a number of restrictions, which should be acknowledged. First off, the SEER database’s lack of essential prognostic factors, such as complex surgical procedures, specialized radiotherapy protocols, precise treatment with chemotherapy regimens, pharmacological interventions, and related information, limited the breadth of our findings regarding patients with LLM. Second, because the dataset only included data from a few US states, the generalizability of our study findings was constrained by the lack of external validation. The DeepSurv model will be improved in the future by adding more, more varied information with a wider geographic reach. Thirdly, the DeepSurv model’s hidden layer’s intrinsic opacity, which functions as a computational “black box,” made it difficult to understand the specific mechanics underlying its ability to forecast the future and the decision-making process it uses. Through thorough study and clarification, we aim to address the aforementioned constraints in our next study [29].

## Conclusion

A deep-learning-driven prognosis model for LLM that is effectively developed will have important therapeutic implications. This model is ready to assist doctors in making informed choices about treatment options, making it easier to identify high-risk patients who may benefit from more aggressive approaches or alternative therapy approaches. Furthermore, accurate survival predictions can encourage patient counseling and team decision-making, encouraging patients and their families to take an active role in their treatment.

### Supplementary Information

**Additional file 1.**

## Data Availability

Publicly available datasets were analyzed in this study. This data can be found at: https://seer.cancer.gov.
